# OTUD7B knockdown inhibits proliferation and autophagy through AKT/mTOR signaling pathway in human prostate cancer cell

**DOI:** 10.1007/s12672-024-01073-2

**Published:** 2024-06-27

**Authors:** Yae Ji Kim, Hui Ju Lee, Kyung Hyun Kim, Sung Pil Cho, Ju Young Jung

**Affiliations:** Department of Veterinary Medicine, Institute of Veterinary Science, College of Veterinary Medicine, Chungnam National University, 220 Gung-Dong, Yusung-Gu, Daejeon, 34134 Republic of Korea

**Keywords:** Autophagy, Prostate cancer, Proliferation, mTOR, OTUD7B

## Abstract

Prostate cancer (PCa) is the second leading disease of cancer-related death in men around the world, and it is almost impossible to treat advanced PCa. OTUD7B is a member of the deubiquitinase family that undergoes a post-translational transformation process, which is essential for cell stability and signaling and is known to play a critical role in cancer. However, its role in PCa has not been discovered. The aim of the study was to investigate the expression and mechanism of OTUD7B in PCa cells. According to the database, high OTUD7B expression showed a poor prognosis. Therefore, we downregulated OTUD7B using siRNA and confirmed the role of OTUD7B in PC3 prostate cancer cells. OTUD7B knockdown effectively induced apoptosis and inhibited the proliferation in PC3 cells. OTUD7B knockdown inhibited autophagy through AKT/mTOR signaling. We also confirmed the relationship between AKT/mTOR signaling and autophagy through rapamycin, an mTOR inhibitor. Taken together, OTUD7B promotes the proliferation, and autophagy, and inhibits apoptosis of prostate cancer cells via the AKT/mTOR signaling pathway.

**Supplementary Information:**

The online version contains supplementary material available at 10.1007/s12672-024-01073-2.

## Introduction

Prostate cancer (PCa) is a common malignancy and the second leading cause of cancer-related deaths in men worldwide [1]. The incidence and mortality rates of prostate cancer rises with age, with 60% incidence and almost 55% of all deaths in men over 65 years [2]. Early-stage PCa can be treated with surgery, radiation, or androgen deprivation [1]. These treatments are initially beneficial; however, treatment for advanced PCa is almost impossible [3, 4]. Thus, it is vital to elucidate PCa-related genes to investigate PCa tumorigenic mechanisms, which will help provide better therapeutic strategies for treatment.

Recent studies have shown a strong correlation between cancer progression and deubiquitination (DUB) levels. DUB is a post-translational modification that is common and important, playing an essential role in cell stability and signaling by hydrolyzing the ubiquitin chains on target proteins [5, 6]. In cancer, dysregulation of deubiquitination processes can lead to aberrant accumulation or activation of oncogenic proteins or the loss of tumor suppressors, contributing to various hallmarks of cancer such as uncontrolled cell proliferation, evasion of apoptosis, genomic instability, and metastasis [7–9]. The deubiquitinase OTUD1 inhibits progression by deubiquitinating and stabilizing KLF4 in non-small cell lung cancer [10]. The deubiquitinase USP35 regulates mitotic progression by modulating the stability of Aurora B [11]. Recently, various reports have shown that DUBs are involved in regulating cancer cell metabolism and have received increasing attention [12–14].

The ovarian tumor domain-containing 7B (OTUD7B) protein belongs to the DUB family and controls many important cellular signaling pathways, such as those associated with inflammation and hypoxia [15, 16]. Recent studies have shown that high OTUD7B expression is closely associated with poor prognosis in several cancers, including breast, lung, and pancreatic cancers [17–20]. OTUD7B inhibits the proliferation and migration of breast cancer cells [17]. OTUD7B accelerated gastric cancer progression by deubiquitizing and stabilizing YAP1 [21]. OTUD7B suppressed lung cancer progression by improving mitochondrial dysfunction [22]. However, it remains unclear whether OTUD7B regulates PCa tumorigenesis. In our study, we explored the role of OTUD7B in PCa cells.

## Material and methods

### Gene expression profiling interactive analysis (GEPIA)

The GEPIA database (http://gepia.cancer-pku.cn/) provided data on 492 tumors and 152 healthy samples from The Cancer Genome Atlas (TCGA) and Genotype-Tissue Expression (GTEx). Gene Expression Profiling Interactive Analysis (GEPIA) was used to analyze differences in OTUD7B expression between healthy tissues and tumor tissues in patients with prostate cancer.

### Cell culture and reagents

Human prostate cancer cell lines (LNCaP and PC3), a human benign prostatic hyperplasia epithelial cell line (BPH-1), and a human normal prostate epithelial cell line (RWPE-1) were purchased from the American Type Culture Collection (ATCC, VA, USA). PC3, LNCaP, and BPH-1 cells were cultured in RPMI-1640 medium (Gibco, NY, USA) supplemented with 10% fetal bovine serum (FBS; Gibco) and 1% antibiotics (Gibco). RWPE-1 cells were cultured in serum-free keratinocyte medium (Gibco). All cells were grown in incubated at 37 °C with 5% CO_2_. Rapamycin was purchased from Sigma-Aldrich (St. Louis, MO, USA). Rapamycin was dissolved in DMSO and diluted in 1640 medium to final concentrations of 100 nM and analyzed after treatment for 24 h.

### siRNA and plasmid transfection assay

The small interfering RNAs (siRNAs) were synthesized by Bioneer (Daejeon, Korea). The sequences of the siRNAs against human OTUD7B were as follows: siOTUD7B; 5′-CCGAGUGGCUGAUUCCUAU-3′. Non-silencing siRNA (siNC) was used as a negative control. siRNA transfection was performed using FuGENE SI transfection reagent (Fugent LLC, Madison, WI, USA) following to the manufacturer’s protocol.

### Cell viability analysis

The cell viability was determined using an Ez-Cytox cell viability assay kit (DoGen, Seoul, Korea) at the indicated time points, according to the manufacturer’s instructions. The cells were seeded in 96-well culture plates (2 × 10^4^ cells/well). PC3 cells were transfected with the indicated siRNAs for 12, 24, 48, and 72 h. Ez-cytox reagent was added 10 μl per well and incubated for 1 h at 37 °C. Absorbance was measured at 450 nm using a microplate reader.

### Colony formation assay

For the clone formation assay, cells were seeded in 6-well plates (5 × 10^3^ cells/well). After 24 h, the cells were transfected with OTUD7B siRNA and cultured in a humidified incubator at 37 °C with 5% CO_2_ for 2 weeks. After 14 d, the cells were fixed with 4% paraformaldehyde. Fixed colonies were stained with 0.5% crystal violet (Sigma, MO, USA) for 20 min at room temperature and analyzed using the ImageJ software (NIH, Bethesda, Maryland, USA).

### Flow cytometry analysis

We seeded PC3 cells in 6 well plates at 2 × 10^5^ cells/well and incubated at 37 °C for 24 h. PC3 cells transfected with either siNC or siOTUD7B for 48 h were analyzed for apoptosis using the FITC annexin V apoptosis detection kit (Biolegend, USA). Transfected cells were centrifuged for 5 min and washed with PBS. The cells were added and resuspended with 100 μl Annexin V binding buffer. Additionally, FITC Annexin V 5 μl and PI solution 10 μl were added and incubated at room temperature in the dark for 10 min. Annexin V binding buffer 400 μl was added and flow cytometry was performed using the FACS Canto II (BD, CA, USA). Annexin V+/PI- (early apoptotic) and Annexin V+/PI+ (late apoptotic) were considered apoptosis.

### Western blot analysis

PC3 cells were lysed with a Radio-Immunoprecipitation Assay buffer (RIPA, Cell Signaling, MA, USA) for 15 min, and centrifuged at 12,000 rpm at 4 °C for 15 min. The supernatant was collected and used for western blot analysis. Samples were separated by 8–12% sodium dodecyl sulfate-polyacrylamide gel electrophoresis (SDS-PAGE). The samples were electronic (Bio-Rad, Hercules, CA, USA) to polyvinylidene fluoride (PVDF) membranes (Millipore, Boston, MA, USA). Then, PVDF membranes were blocked with 5% skim milk in PBS-T for 2 h. Membranes were incubated at 4 °C overnight with the following primary antibodies: anti-OTUD7B (Proteintech, Wuhan, China), anti-ATG7, anti-caspase 3, anti-p62 (Santa Cruz, TX, USA), anti-PARP, anti-p-AKT (Ser473), anti-AKT (Cell Signaling), anti-p-mTOR (Ser2448), anti-mTOR (GeneTex, CA, USA), anti-VPS34, anti-ATG14L, anti-p-Becline1 (Ser234), and anti-LC3B, anti-caspase 9, anti-β-actin (Abcam, Cambridge, UK), and anti-glyceraldehyde 3-phosphate dehydrogenase (GAPDH, CityLab, USA) at 1:1000 dilution. After 24 h, the membranes were washed three times for 5 min each. Membranes were then incubated with horseradish peroxidase (HRP)-conjugated IgG secondary antibody (anti-rabbit or anti-mouse, 1:5000, GenDEPOT, TX, USA) at RT for 2 h. Protein bands were detected using a chemiluminescent detection reagent (Bio-Rad). Analysis was performed using a CS Analyzer 4 (ATTO, Tokyo, Japan).

### Immunofluorescence staining

Transfected PC3 cells were fixed with 4% paraformaldehyde for 10 min and washed wish PBS for 5 min, and blocked with 3% bovine serum albumin for 30 min at RT. Cells were then incubated with LC3B (1:100, Cell signaling) for 1 h at RT. The cells were washed twice with PBS for 5 min and incubated with goat anti-rabbit HRP peroxidase-conjugated IgG secondary antibodies for 30 min at RT. The nucleus was counterstained with 4′6-diamino-2-phenyliondole (DAPI, Vector Labs, CA, USA). Images were acquired using an Eclipse 80i microscope (Nikon, Tokyo, Japan).

### Statistical analysis

All experiments were conducted in a double-blind manner. The results of each experiment are expressed as the mean ± standard deviation (SD). One-way ANOVA was used to compare two or more groups, followed by a Tukey post hoc test. Statistical and graphical analyses were carried out with PRISM version 5 (GraphPad Software, CA, USA).

## Results

### OTUD7B is upregulated in prostate cancer cells

**Fig. 1:**
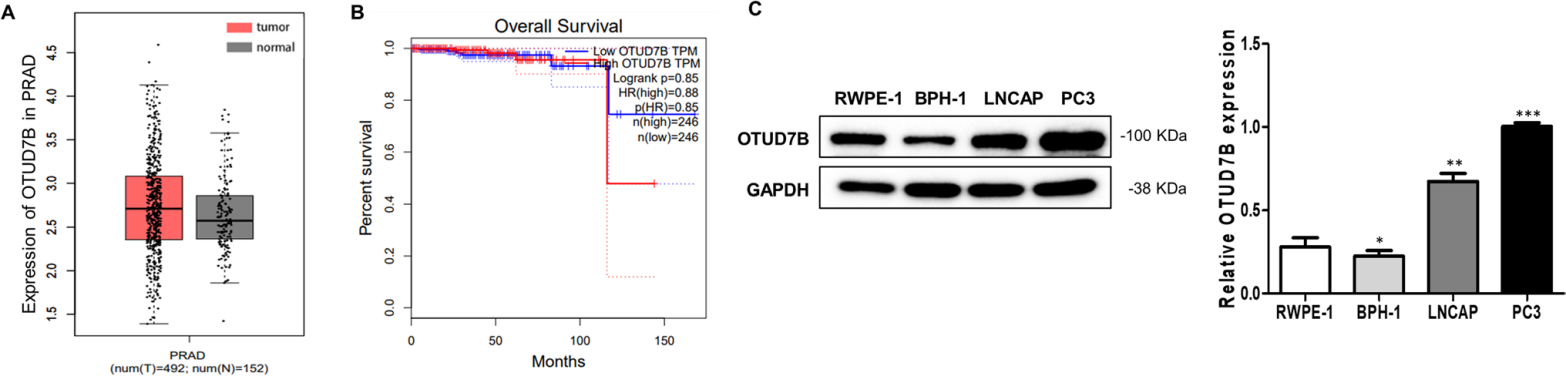
OTUD7B expression levels in prostate cancer. **A** The prostate cancer compared with normal tissues in the GEPIA database. **B** Overall survival based on Kaplan–Meier curve of OTUD7B expression; high OTUD7B expression group (red line) was lower than that of low OTUD7B expression group (blue line). **C** Expression of OTUD7B in prostate normal epithelial cell lines (RWPE-1, BPH-1) and prostate cancer cell lines (LNCaP, PC3). Data is represented as mean ± SD. *p < 0.05, **p < 0.01, ***p < 0.001

According to the GEPIA database, OTUD7B has a higher expression in tumor prostate tissue (T) compared to normal prostate tissue (N) and is associated with poor prognosis in prostate cancer patients (Fig. 1A and B). Furthermore, we measured the protein expression of OTUD7B in RWPE-1, BPH-1, LNCaP, and PC3 cells using western blotting. OTUD7B expression was significantly elevated in prostate cancer cell lines, especially in PC3 cells (Fig. 1C). Similarly, in LNCAP cells, the expression of OTUD7B significantly increased, but there was no significant change in cell viability and apoptosis rate (Supplementary Fig. 1A and B). Hence, we were selected PC3 cells for the study of the OTUD7B in PCa.

### OTUD7B knockdown inhibits cell proliferation and induces apoptosis in PC3 cells

**Fig. 2:**
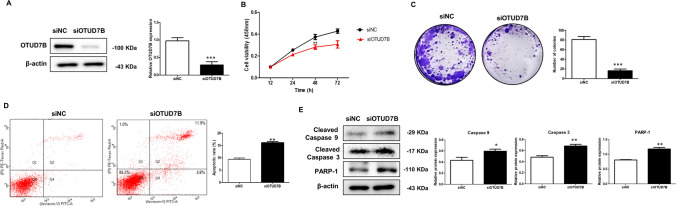
OTUD7B knockdown inhibits cell proliferation and induces apoptosis in PC3 cells. **A** PC3 cells were transfected with siRNA of OTUD7B for 48 h. **B** Cell proliferation of OTUD7B knockdown cells. **C** Proliferative ability of OTUD7B knockdown as determined by colony formation assay. **D** FACS analysis of annexin V/PI staining. **E** Levels of apoptosis-related factors such as cleaved caspase9, cleaved caspase3, and PARP-1 in OTUD7B knockdown cells. Data is expressed as the mean ± SD. siNC: control transfected siRNA; siOTUD7B: OTUD7B transfected siRNA. *p* < 0.05, **p < 0.01, ***p < 0.001

To investigate the effect of OTUD7B on PC3 cells, we examined the biological functions of OTUD7B in cell proliferation and apoptosis in these cells. siOTUD7B construct was transfected into PC3 cells for siRNA treatment (Fig. 2A). The cell viability assay results showed that OTUD7B knockdown significantly gradually decreased cell proliferation over time compared to negative control cells (Fig. 2B). The colony forming assay results showed that OTUD7B knockdown significantly decreased the clonogenic ability of PC3 cells (Fig. 2C). Additionally, OTUD7B knockdown was found to induce apoptosis in PC3 cells. OTUD7B knockdown increased the apoptosis rate, as shown by annexin V/PI staining and the expression of cleaved-caspase 9, cleaved-caspase 3, and PARP-1 (Fig. 2D and E). These results show that OTUD7B downregulation greatly inhibits cell proliferation and promotes apoptosis in PC3 cells.

### OTUD7B knockdown inhibits autophagy in prostate cancer cells via the AKT/mTOR signaling pathway

**Fig. 3:**
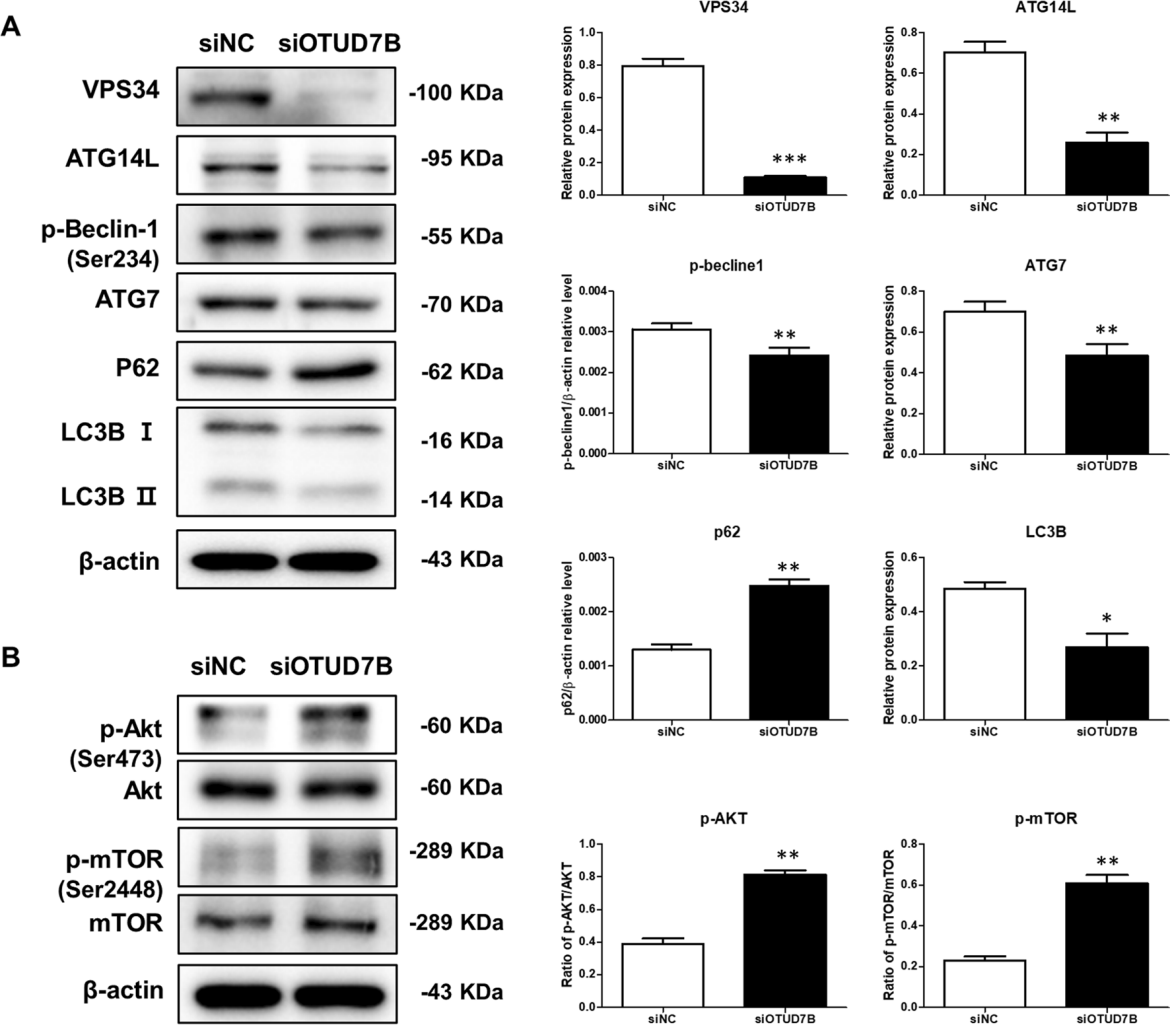
OTUD7B knockdown inhibits autophagy in prostate cancer cells via the AKT/mTOR signaling pathway. **A** Expression levels of VPS34, ATG14L, p-Becline1, ATG7, p62, and LC3B in the PC3 cell treated with siNC or siOTUD7B. **B** Expression levels of p-mTOR, mTOR p-AKT, and AKT. Data were expressed as the mean ± SD. siNC: control transfected siRNA; siOTUD7B: OTUD7B transfected siRNA. *p* < 0.05, **p < 0.01, ***p < 0.001

We investigated whether OTUD7B regulated autophagy in PC3 cells. As shown in Fig. 3A, our results showed that OTUD7B knockdown significantly decreased the levels of VPS34, ATG14L, p-Becline1, ATG7, and LC3B-II/I, while levels of P62 were significantly increased compared with the negative control cell. OTUD7B knockdown significant increased the level of p-Akt and p-mTOR (Fig. 3B). Overall, our results suggest that OTUD7B knockdown reduces autophagy by activating AKT/mTOR signaling in PC3 cells.

### OTUD7B regulated autophagy following rapamycin treatment

**Fig. 4:**
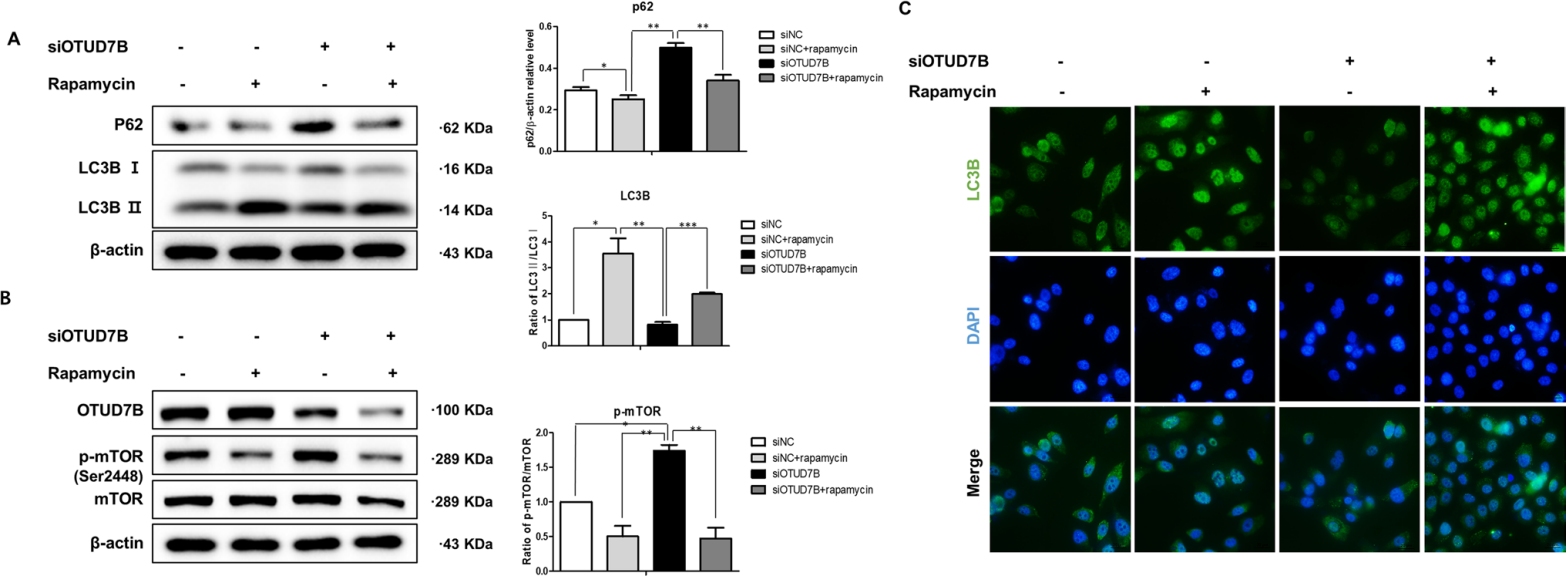
OTUD7B regulated autophagy following rapamycin treatment. **A** Expression levels of p62 and LC3B in siNC or siOTUD7B treated with rapamycin. **B** Expression levels of p-mTOR and mTOR. **C** Fluorescent staining for the detection of LC3B. siNC or siOTUD7B treated with 100 mM rapamycin were labeled with anti-LC3B and imaged by microscopy. Images are at ×400 magnification. Data were expressed as the mean ± SD. *p* < 0.05, **p < 0.01, ***p < 0.001

Rapamycin affects autophagy as an inhibitor of mTOR that regulates mTOR expression [23]. Next, we reconfirmed the activity of mTOR and the effect of autophagy by treating rapamycin. LC3B-II/I expression was increased and p62 and p-mTOR expression was decreased in the group treated with 100 nM rapamycin in OTUD7B knockdown cells (Fig. 4A and B). In addition, the immunofluorescence distribution pattern of LC3B was also shown to be significantly increased in the rapamycin-treated group (Fig. 4C).

## Discussion

Prostate cancer (PCa) is the common cancers in men, and the second leading cause of cancer-related deaths in the United States [24]. Despite the rapid development of various treatments, treating advanced PCa is almost impossible [25]. Therefore, the clinical application of PCa-targeting therapies has become an important focus in PCa research.

Notably, DUB is closely associated with cancer progression. OTUD7B, a member of the DUB family, acts on various cancers, such as breast, pancreatic, and lung cancers. OTUD7B is associated with poor prognosis and promotes cell proliferation in cancer. Jianing Tang et al. reported that OTUD7B induces tumorigenesis through ERα protein expression in breast cancer [17]. Chen et al. reported that OTUD7B promotes proliferation, migration, and invasion by stabilizing N1ICD in pancreatic cancer [20]. Additionally, OTUD7B promotes tumor growth through the AKT/VEGF signaling pathway in lung adenocarcinoma [26]. However, the role of OTUD7B in PCa cancer remains unclear. Therefore, we investigated the role of OTUD7B in PCa. According to the GEPIA database, OTUD7B expression is higher in prostate tumor tissues than in healthy tissues. Furthermore, high OTUD7B expression is associated with poor prognosis in patients with PCa, which in turn is associated with abnormal proliferation of cancer [27]. In our study, the expression of OTUD7B was significantly increased in human prostate cancer cell lines compared to that in normal prostate cell lines, especially PC3 cells. OTUD7B affects the proliferation of PC3 cells. OTUD7B knockdown gradually reduced the cell viability rate over time. Cell proliferation plays a crucial role in prostate cancer development and progression [28]. These results suggest that OTUD7B significantly increases prostate cancer cell proliferation.

Apoptosis is a fundamental biological process crucial in maintaining tissue homeostasis and regulating cell growth. Apoptosis is a natural defense mechanism against cancer development and progression [29]. In PCa, the ability of cancer cells to evade apoptosis is a key factor contributing to tumor growth [30]. Substantial and long-term stress induces apoptosis accompanied by caspase activity. Many studies have reported that increased caspase 3 and PARP-1 expression induces apoptosis in PCa [31, 32]. In this study, OTUD7B knockdown increased the protein levels of apoptotic factors, such as cleaved caspase-3, cleaved caspase-9, and PARP-1 in PC-3 cells. These results indicated that OTUD7B knockdown promoted apoptosis in PC3 cells. However, the apoptosis is not large, so OTUD7B knockdown lead to minimal increase in apoptosis.

Autophagy is a cellular process in which unnecessary and dysfunctional organelles in cells are degraded and recycled [33]. In cancer, autophagy plays a dual role as a tumor suppressor and a cell survival mechanism [31, 34]. Autophagy is thought to regulate apoptosis in tumor cells [35]. Caspases can break down and digest the proteins needed for autophagy, disable autophagy, disrupt cell protection functions, and promote cell death [36]. The AKT/mTOR signaling pathway is a critical pathway for controling autophagy [37]. mTOR, which acts downstream of AKT, plays an important role in regulating PCa by acting as a functional mediator [38]. A previous study found that MiR-146b inhibits autophagy in PCa via the PTEN/Akt/mTOR signaling pathway [39]. Furthermore, Wang et al. reported that OTUD7B was associated with mTOR signaling in lung cancer [40]. OTUD7B lowers the ubiquitous levels of GbL, which determines the homeostasis of mTORC2 formation and activation, induces mTORC2/AKT signaling pathway activation, and downregulates mTORC1 expression in vitro. To identify the underlying mechanism, we speculated that OTUD7B knockdown inhibits autophagy via the AKT/mTOR signaling pathway in PCa cells. Our results showed increased protein levels of p-AKT and p-mTOR following OTUD7B siRNA transfection and decreased conversion of LC3-I to LC3-II. In addition, OTUD7B knockdown significantly decreased levels of the autophagosome formation markers VPS34, ATG14L, and ATG7 and significantly increased p62. Furthermore, we confirmed the activation of the OTUD7B pathway following rapamycin treatment, which is an mTOR inhibitor. In the rapamycin treatment group, we confirmed decreased p-mTOR expression and increased autophagy-related factor expression. These findings lead us to suggest that the role of OTUD7B in prostate cancer cells may be linked to autophagy through the modulation of the mTOR signaling pathway.

Collectively, our current study demonstrated that OTUD7B knockdown inhibits autophagy through activating AKT/mTOR pathway and positively contributes to the extrinsic apoptosis and proliferation of prostate cancer cell.

## Conclusions

In conclusion, this study suggests that OTUD7B is closely related to prostate cancer progression. OTUD7B is upregulated in prostate cancer cell lines, and its knockdown inhibits proliferation, and autophagy and promotes apoptosis in PC3 cells. In addition, OTUD7B may act by activating the AKT/mTOR signaling pathway. Although the detailed molecular mechanism is still unclear, our findings suggest that OTUD7B plays an important role in prostate cancer progression and may be a potential therapeutic target for the treatment of prostate cancer.

### Supplementary Information

Below is the link to the electronic supplementary material.

Supplementary file 1 (PDF 784 KB)

## Data Availability

The data used to support the findings of this study are available from the Gene Expression Profiling Interactive Analysis database (http://gepia.cancer-pku.cn/). All data generated or analyzed during this study are included in this published article.
