# Supplementary material for: OTUD7B knockdown inhibits proliferation and autophagy through AKT/mTOR signaling pathway in human prostate cancer cell
Source: Discov Oncol. 2024 Jun 27;15:247. doi: 10.1007/s12672-024-01073-2 (PMC11211289; doi:10.1007/s12672-024-01073-2)
Supplement: Supplementary file 1 — (PDF 784 KB) [file 12672_2024_1073_MOESM1_ESM.pdf]

OTUD7B knockdown inhibits proliferation and autophagy through AKT/mTOR signaling pathway in human prostate cancer cell

Fig1.C-OTUD7B

Manuscript image

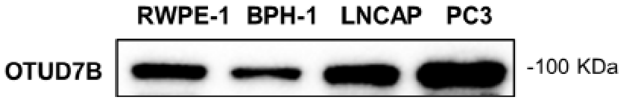

Blot image

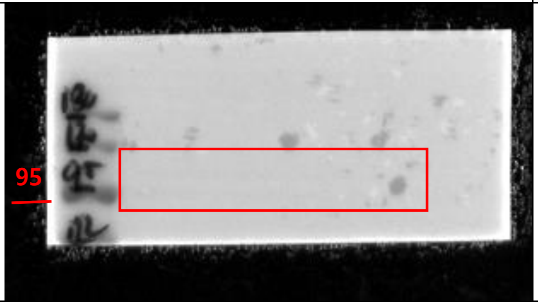

Merge image

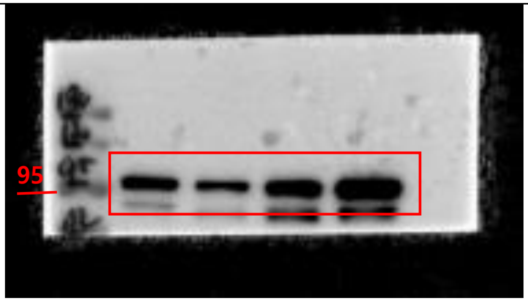

Fig1.C-GAPDH

Manuscript image

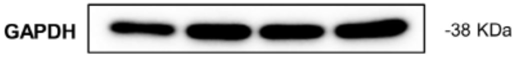

Blot image

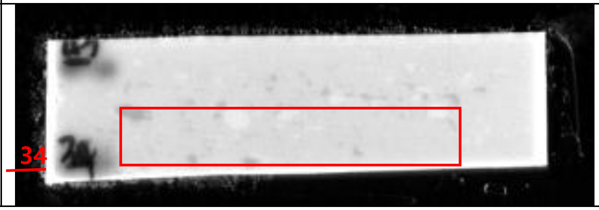

Merge image

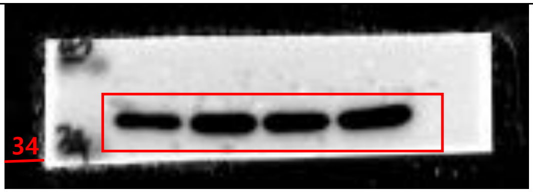

Fig2.A- OTUD7B

Manuscript image

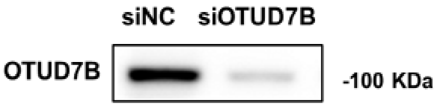

Blot image

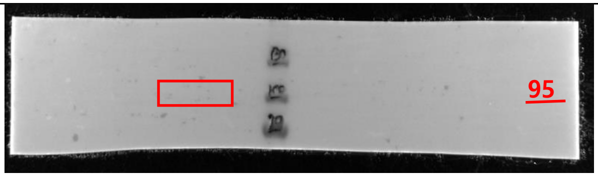

Merge image

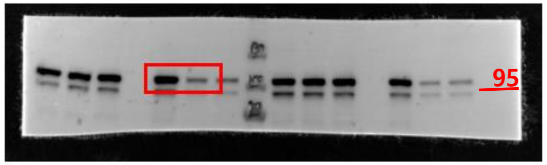

Fig2.A- β-actin

Manuscript image

Blot image

Merge image

β-actin 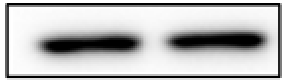 -43 KDa	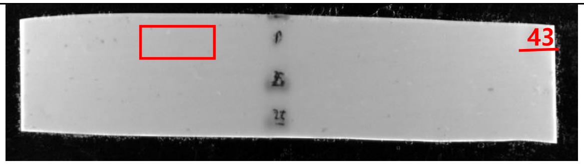	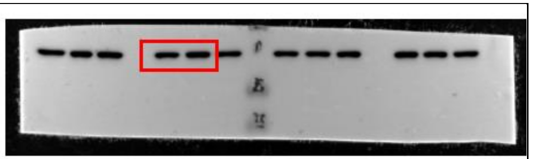
**Fig2.E-** Caspase9		
Manuscript image	Blot image	Merge image
siNC siOTUD7B<br>Cleaved Caspase 9 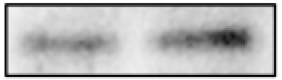 -29 KDa	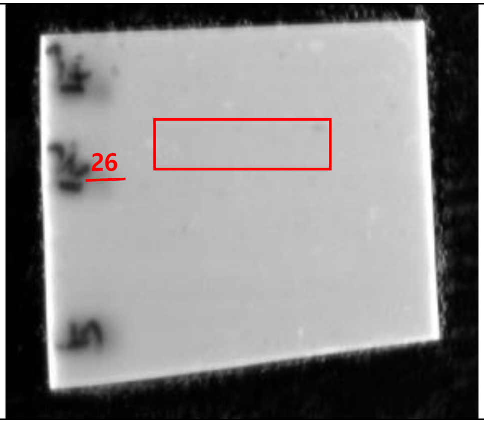	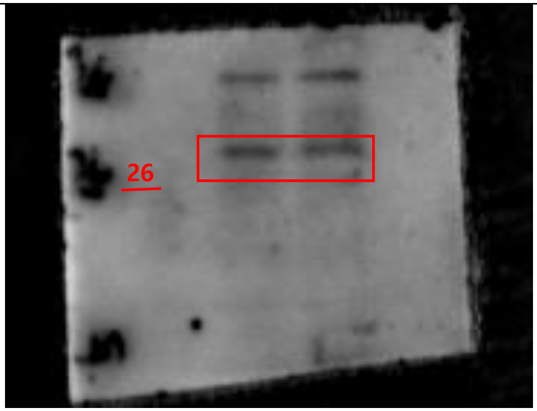
**Fig2.E-** Caspase3		
Manuscript image	Blot image	Merge image
Cleaved Caspase 3 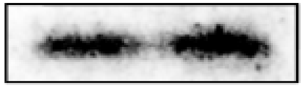 -17 KDa	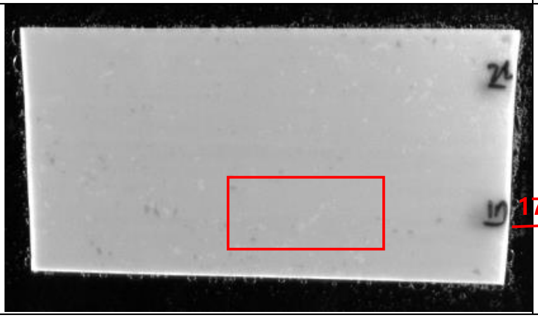	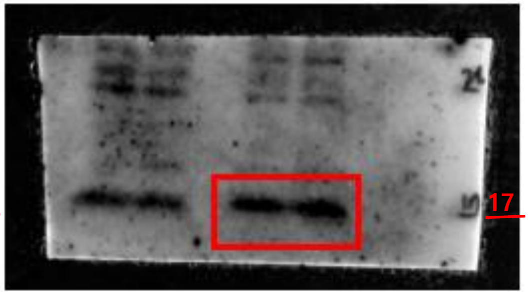
**Fig2.E-** PARP-1		
Manuscript image	Blot image	Merge image

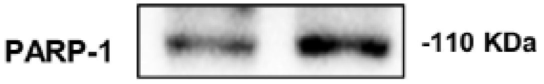	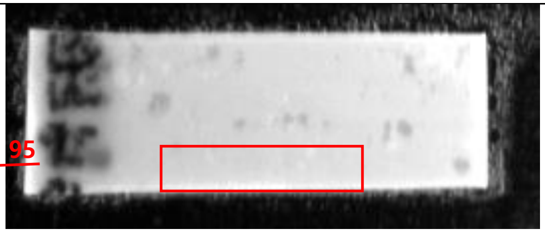	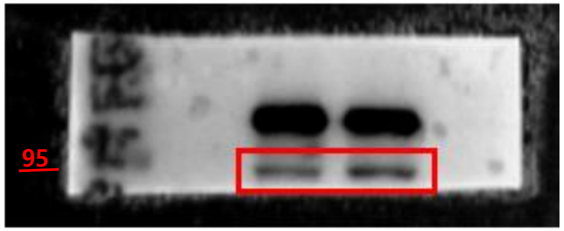
**Fig2.E-** β-actin		
Manuscript image	Blot image	Merge image
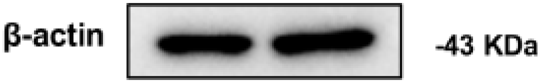	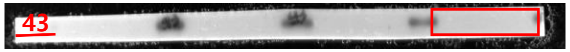	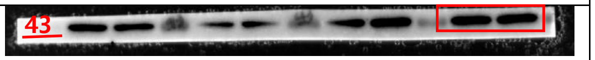
**Fig3.A-** VPS34		
Manuscript image	Blot image	Merge image
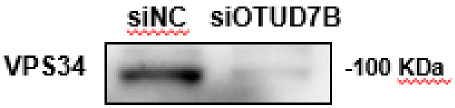	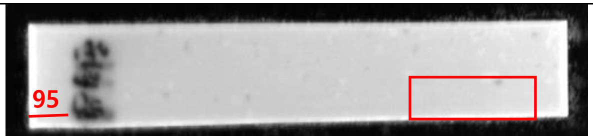	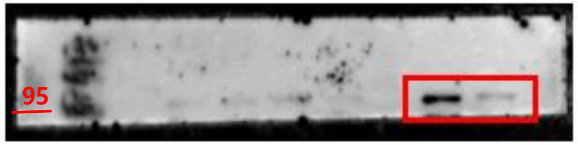
**Fig3.A-**ATG14L		
Manuscript image	Blot image	Merge image
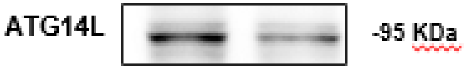	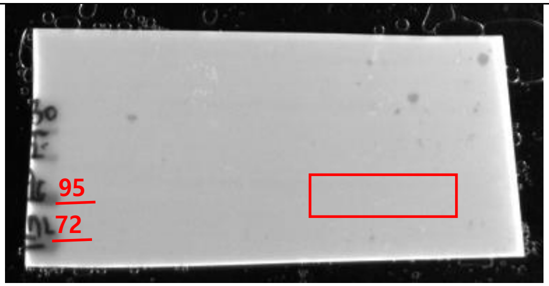	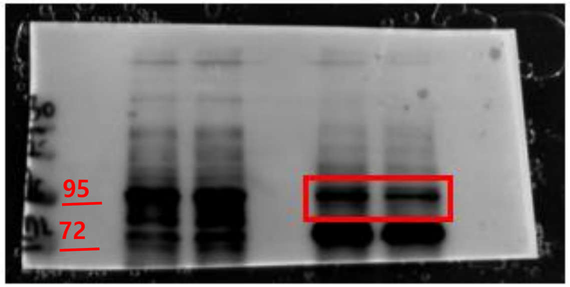

**Fig3.A-**p-Beclin-1		
Manuscript image	Blot image	Merge image
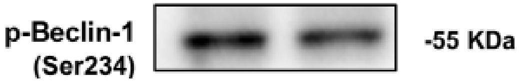	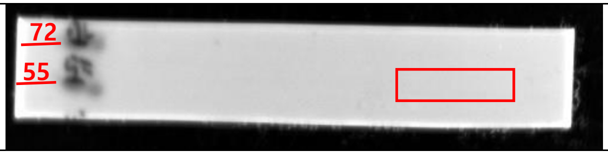	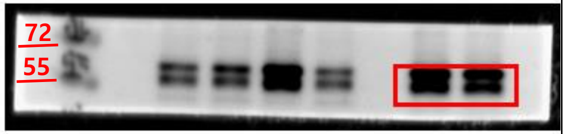

**Fig3.A-**ATG7		
Manuscript image	Blot image	Merge image
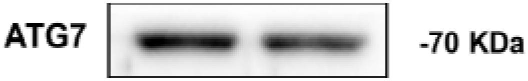	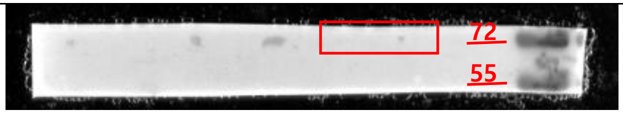	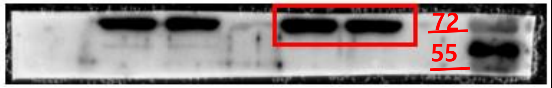

**Fig3.A-**P62		
Manuscript image	Blot image	Merge image
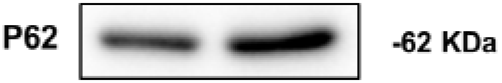	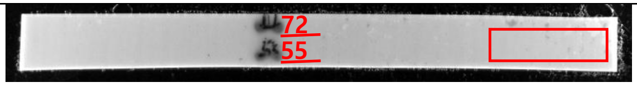	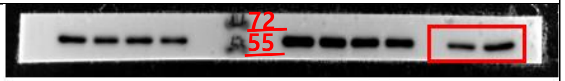

**Fig3.A-**LC3B		
Manuscript image	Blot image	Merge image
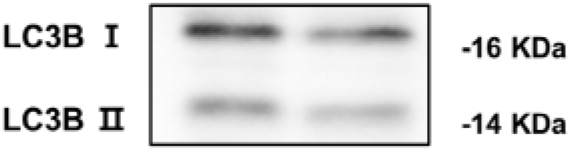	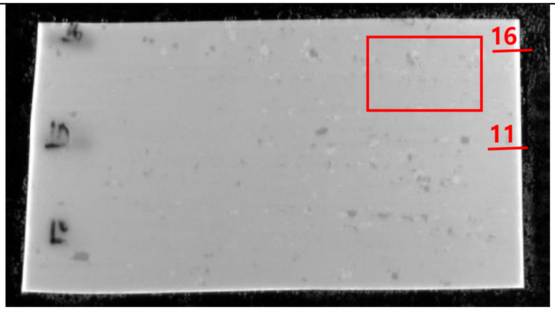	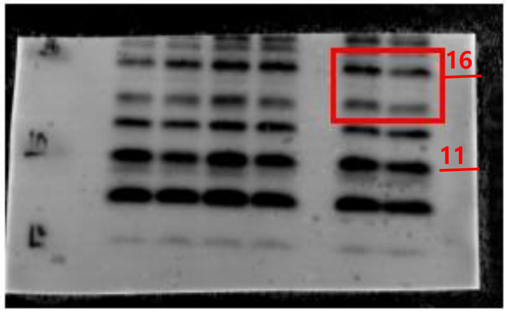

<table>
<tr><td colspan="3">Fig3.A-β-actin</td></tr>
<tr><td>Manuscript image</td><td>Blot image</td><td>Merge image</td></tr>
<tr><td>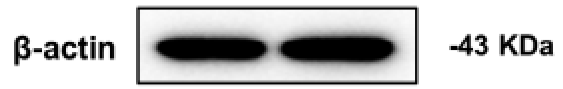
</td><td>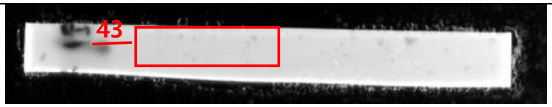
</td><td>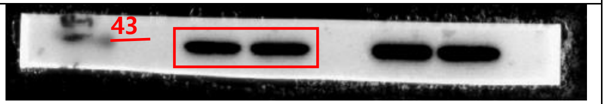
</td></tr>
<tr><td colspan="3">Fig3.B-p-AKT</td></tr>
<tr><td>Manuscript image</td><td>Blot image</td><td>Merge image</td></tr>
<tr><td>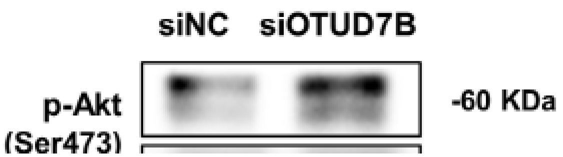
</td><td>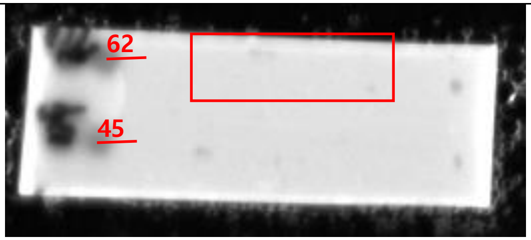
</td><td>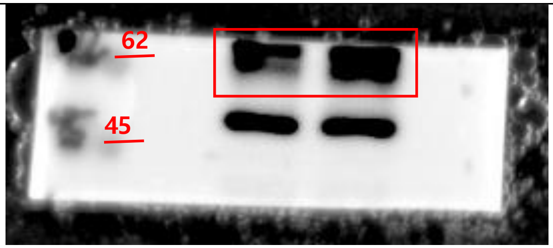
</td></tr>
<tr><td colspan="3">Fig3.B-AKT</td></tr>
<tr><td>Manuscript image</td><td>Blot image</td><td>Merge image</td></tr>
<tr><td>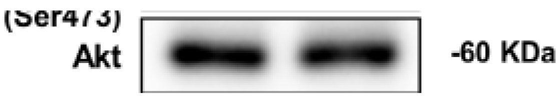
</td><td>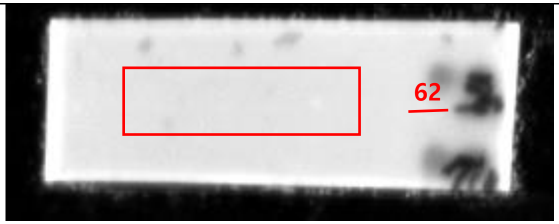
</td><td>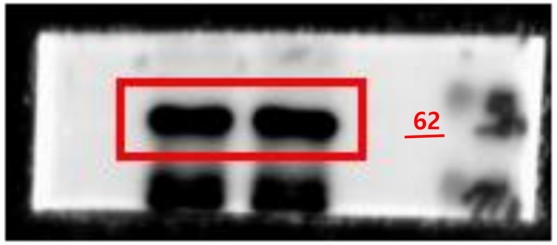
</td></tr>
<tr><td colspan="3">Fig3.B-p-mTOR</td></tr>
<tr><td>Manuscript image</td><td>Blot image</td><td>Merge image</td></tr>
</table>

p-mTOR (Ser2448) 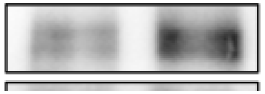 -289 KDa	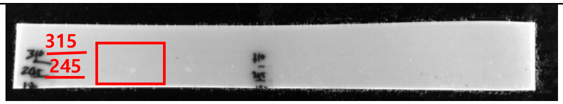	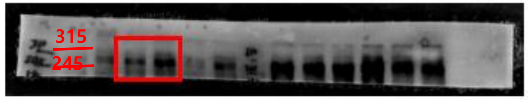
**Fig3.B-**mTOR		
Manuscript image	Blot image	Merge image
mTOR 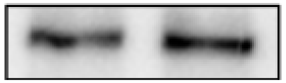 -289 KDa	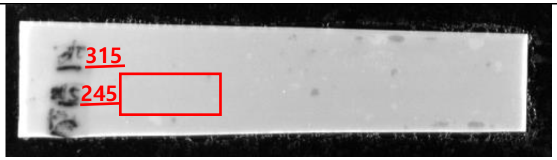	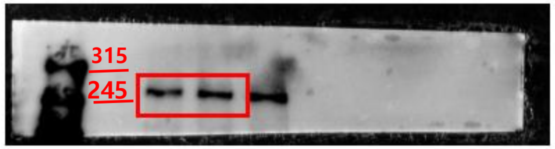
**Fig3.B-**β-actin		
Manuscript image	Blot image	Merge image
β-actin 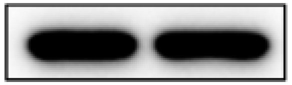 -43 KDa	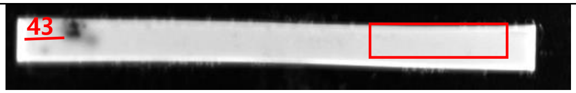	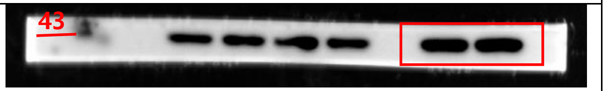
**Fig4.A-**P62		
Manuscript image	Blot image	Merge image
P62 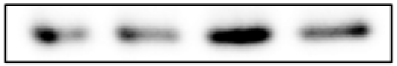 ·62 KDa	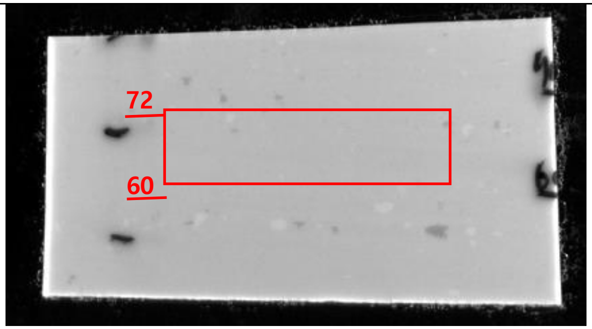	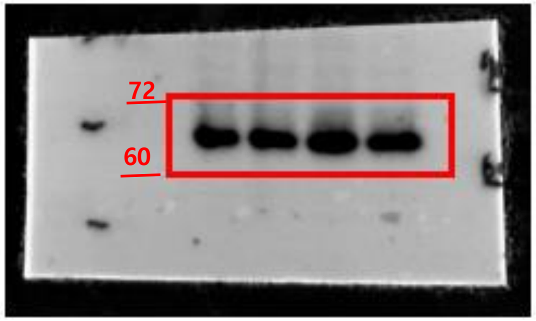

Fig4.A-LC3B

Manuscript image

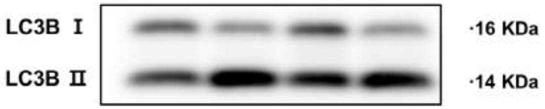

Blot image

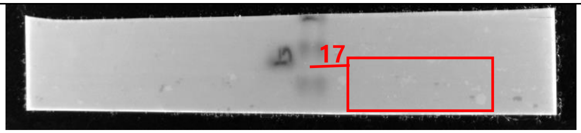

Merge image

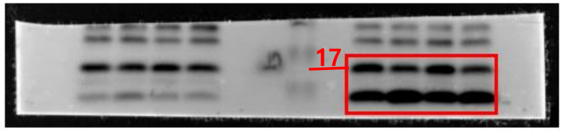

Fig4.A-β-actin

Manuscript image

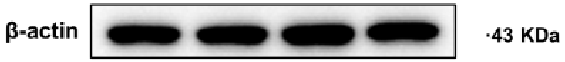

Blot image

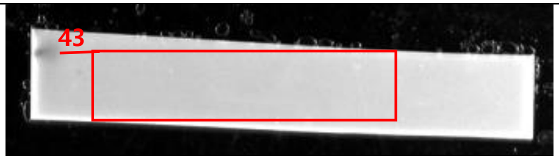

Merge image

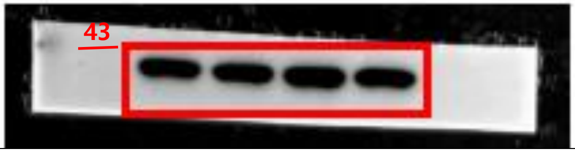

Fig4.B-OTUD7B

Manuscript image

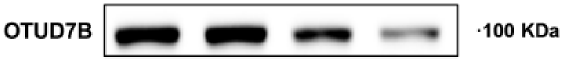

Blot image

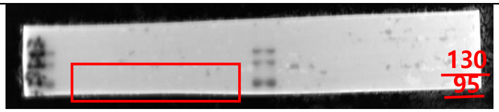

Merge image

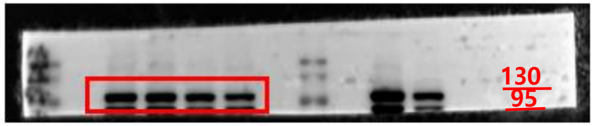

Fig4.B-p-mTOR

Manuscript image

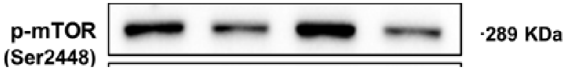

Blot image

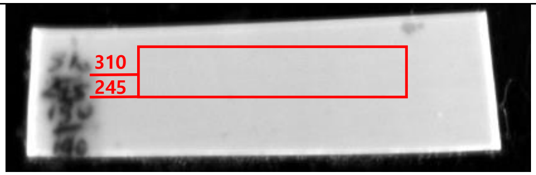

Merge image

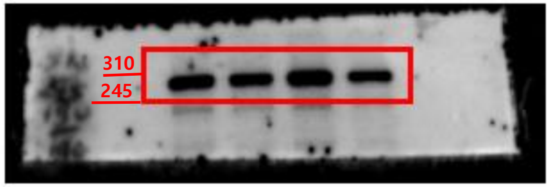

Fig4.B-mTOR

Manuscript image	Blot image	Merge image
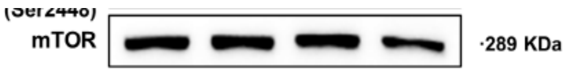	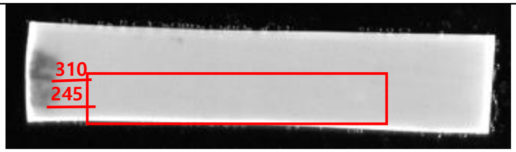	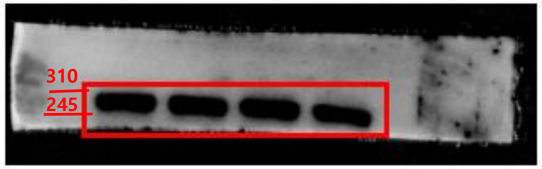
**Fig4.B-**β-actin		
Manuscript image	Blot image	Merge image
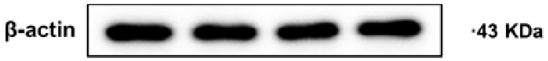	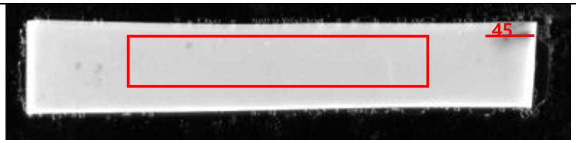	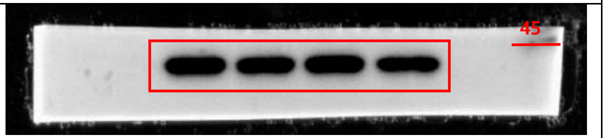
